# Resources and predation: drivers of sociality in a cyclic mesopredator

**DOI:** 10.1007/s00442-022-05107-w

**Published:** 2022-02-03

**Authors:** Rasmus Erlandsson, Malin Hasselgren, Karin Norén, David Macdonald, Anders Angerbjörn

**Affiliations:** 1 Department of Zoology, Stockholm University, 106 91 Stockholm, Sweden; 2 Department of Arctic Ecology-Tromsø, Norwegian Institute for Nature Research, Tromsø, Norway; 3 Wildlife Conservation Research Unit, Department of Zoology, The Recanati-Kaplan Centre, University of Oxford, Tubney House, Abingdon Road, Tubney, OX13 5QL UK

**Keywords:** Cooperative defence, Group-living, Group size, Intra-guild predation, Resource dispersion

## Abstract

In socially flexible species, the tendency to live in groups is expected to vary through a trade-off between costs and benefits, determined by ecological conditions. The Resource Dispersion Hypothesis predicts that group size changes in response to patterns in resource availability. An additional dimension is described in Hersteinsson’s model positing that sociality is further affected by a cost–benefit trade-off related to predation pressure. In the arctic fox (*Vulpes lagopus*), group-living follows a regional trade-off in resources’ availability and intra-guild predation pressure. However, the effect of local fluctuations is poorly known, but offers an unusual opportunity to test predictions that differ between the two hypotheses in systems where prey availability is linked to intra-guild predation. Based on 17-year monitoring of arctic fox and cyclic rodent prey populations, we addressed the Resource Dispersion Hypothesis and discuss the results in relation to the impact of predation in Hersteinsson’s model. Group-living increased with prey density, from 7.7% (low density) to 28% (high density). However, it remained high (44%) despite a rodent crash and this could be explained by increased benefits from cooperative defence against prey switching by top predators. We conclude that both resource abundance and predation pressure are factors underpinning the formation of social groups in fluctuating ecosystems.

## Introduction

Group size and social organisation can vary remarkably in carnivores (Kleiman and Eisenberg 1973; Macdonald 1983). Ecological, phylogenetic, and evolutionary factors influence social organisation, from solitary life to social groups, where some species seem to be invariably solitary or group-living [e.g., the European lynx (*Lynx lynx*) vs African wild dog (*Lycaon pictus*)], whereas others are flexible (the wolf, *Canis lupus*) (Bekoff et al. 1984). Studies of socially flexible species provide an opportunity to investigate the mechanisms affecting group size and, on a longer timescale, those underlying the evolutionary processes that could affect a species position on a continuum from fixed to flexible social structures.

The Resource Dispersion Hypothesis (Macdonald 1983, hereafter RDH) argues that spatio-temporal variation in resource availability is a strong driver for variation in group size in territorial carnivores. The crux of the RDH is that groups may develop where resources are dispersed, such that the smallest economically defensible territory for a pair can also sustain additional animals. Territories are envisaged to be configured, such that they encompass sufficient foraging patches to sustain their primary occupants (e.g., a mated pair) during periods when resource availability is low. During feeding periods, or seasons, when the territory offers food beyond the requirements of the minimal social unit (e.g., a pair), an optimal territory would be smaller. However, where contracting the territory is not feasible, an alternative is to accept additional occupants within the existing territorial borders. In other words, the territory size of what Carr and Macdonald (1986) term the ‘primary occupants’ (e.g., a territorial pair) would be determined by the ‘bottleneck’ conditions, but the cost of accepting ‘secondary occupants’ (called joiners by Brown 1982) to share a territory would be negligible under the more plentiful conditions (Macdonald 1983; Carr and Macdonald 1986). Group size in species living under fluctuating resource conditions therefore provides particularly insightful circumstances to test RDH.

From the perspective of RDH, formation of groups can occur without cooperation or other sociological benefits to members (Macdonald and Carr 1989; Macdonald and Johnson 2015). However, within the framework of RDH, some cooperative benefits may also occur (see also Kruuk and Macdonald 1985). The RDH is often invoked in the context of the Carnivora, but the principles would apply to any taxon, including mesopredators and prey species, where cooperative defence against predators would be a benefit of group-living (Pulliam 1973). Following the early work of (Kruuk et al. 1979) on badgers, early explorations of the RDH focused on the red fox (Macdonald 1981) and then extended to arctic foxes (*Vulpes lagopus*) by Hersteinsson and Macdonald (1982) (see also Hersteinsson 1984). Building on the predictions of the RDH, the Hersteinsson’s model includes variation in both prey abundance and predator guild composition and considered the importance of predation as a mechanistic driver to maintain group-living (Hersteinsson's model, Norén et al. 2012). The model predicts that high predation pressure would increase the tolerance of joiners to capitalise on the advantages of group size. The group size might then increase with high predation pressure even when resource availability (food security in the vocabulary of RDH) remained constant, as the rewards of cooperation counter-balanced the costs of cohabitation. The cost of joiners would be compensated by their contribution to the defence of territory and juveniles: the net outcome would thus favour increased group size (Elmhagen et al. 2013). While the original RDH is based on spatio-temporal dynamics, the Hersteinsson’s model has previously been tested only on spatial variation in predation pressure, disregarding temporal variation (Norén et al. 2012).

Taken together, group size would then be determined by residents having a tolerance for joiners in relation to available resources (Carr and Macdonald 1986). The trade-off would, however, be different for residents and joiners, and group-living should, therefore, be seen in relation to the different roles of individuals in the social group (Brown 1982; Koenig et al. 1992). From the perspective of joiners, sharing a territory would be an alternative to a risky and costly dispersal (Macdonald and Carr, 1989; Koenig et al. 1992; Komdeur 1992). For individuals without a territory, amongst the benefits of joining an established group could be a chance to inherit the territory (Carr and Macdonald 1986; Koenig et al. 1992; Komdeur 1992). For residents, many benefits of group-living could be achieved by sharing territories with any conspecific; however, the gain would be enhanced by the impact of relatedness on inclusive fitness (Hamilton 1964; Akçay and Van Cleve 2016), and this benefit could also affect territory inheritance (Lindström 1986; Koenig et al. 1992). For a comprehensive understanding of group formation in territorial carnivores, it is thus important to consider several factors in parallel.

In the arctic fox, group-living is flexible (Kruchenkova et al. 2009; Norén et al. 2012), making it a revealing model species to investigate mechanisms underlying sociality. Previous large-scale studies on different arctic fox populations support the Hersteinsson’s model (Norén et al. 2012; Elmhagen et al. 2013), showing that the cost–benefit balance of group-living can be explained by regional differences in herbivore prey richness in combination with intra-guild predation pressure [intra-guild predation is here after defined in a broader sense (Polis et al. 1989), including larger terrestrial predators and eagles]. In arctic fox populations driven by cyclic small rodent prey (Angerbjörn et al. 1995), high rodent abundance is not only associated with readily available food but also with a low intra-guild predation pressure from larger predators (Elmhagen et al. 2013; Erlandsson et al. 2017) as the small rodent community (cyclic species of *Arvicolinae)* constitutes a basal prey for all mammal predators and birds of prey in the ecosystem (Murdoch 1969; Nyström et al. 2006; Hellström et al. 2014). However, whereas the increasing phase of small rodent populations is slow, the decrease phase is steep, forming a crash (Turchin et al. 2000). The intra-guild predation pressure on alternative prey, herbivores and mesopredators, will therefore increase drastically and larger predators may actually also switch to arctic foxes. The contrasting ecological conditions throughout the small rodent population cycle can therefore be seen as a natural experiment with temporal variation in both resource availability (rodent prey) and intra-guild predation pressure from prey switching larger predators that actively prey on arctic fox (e.g., golden eagle *Aquila chrysaëtos*, white-tailed eagle *Haliaeetus albicilla*, red fox *Vulpes vulpes*, and wolverine *Gulo gulo*), providing an opportunity to investigate the marginal cost and gains of group-living.

**Fig. 1:**
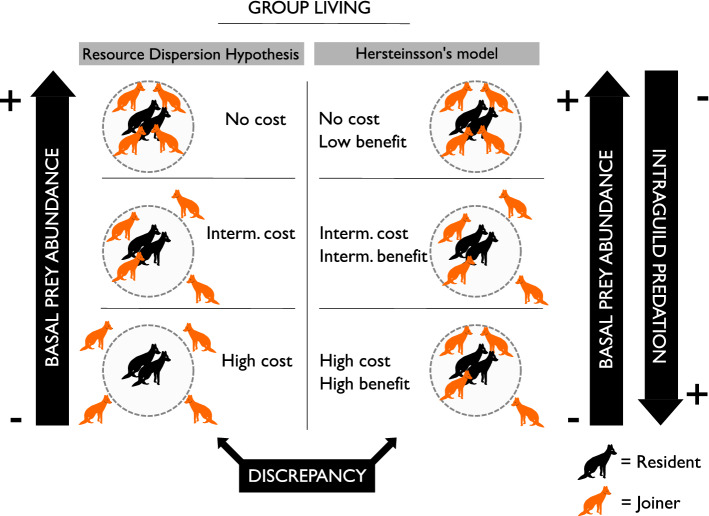
The resource dispersion hypothesis (Macdonald 1983) predicts that high resource abundance increases group-living, and Hersteinsson’s model predicts that high predation pressure increases group-living in prey species with cooperative defence (Norén et al. 2012). In the Scandinavian mountain tundra, top predators switch to alternative prey when basal prey decrease, forming a negative link between food availability and intra-guild predation pressure on mesopredators. The two perspectives have different predictions regarding group size when resources are scarce, but intra-guild predation pressure is high. Hersteinsson’s model has not previously been tested regarding to temporal variation in predation pressure

While resource abundance and predation pressure are both linked to the phase of the rodent cycle, they also tend to be inversely related to each other. This results in contrasting predictions regarding the frequency of group-living during certain phases of the rodent cycle (Fig. 1). During years of low or increasing rodent abundance, we predict low tolerance for joiners, since both resource availability and predation pressure are moderate. During peak years, however, the cost of tolerance should be negligible and group-living common due to abundance of food, although the benefit of cooperative defence likely remains limited. During a rodent decrease or crash, however, the resources for the arctic fox diminish, while the intra-guild predation pressure from top predators would increase (Erlandsson et al. 2017). We would therefore expect that tolerance for joiners would decrease due to stronger competition, while simultaneously the benefit of cooperative defence would increase the tolerance. Depending on which factor is the most important, we would expect a decline in group-living due to high cost for tolerance or, if the marginal gains of cooperative defence are high enough, a large proportion of group-living.

In this study, we used 17 years of breeding census data from a Swedish arctic fox subpopulation to test if group-living varied in relation to temporal fluctuations in prey abundance, and if group-living was more common in territories with higher primary productivity. Furthermore, using proxies for predation pressure, we discuss if temporal changes in intra-guild predation pressure could explain changes in groups under specific phases of the rodent cycle. To test if close relatives were more likely to form social groups, we also used genetic analyses to investigate the relationship between adults when litters were reared by more than two parents.

## Method

### Study area

**Fig. 2:**
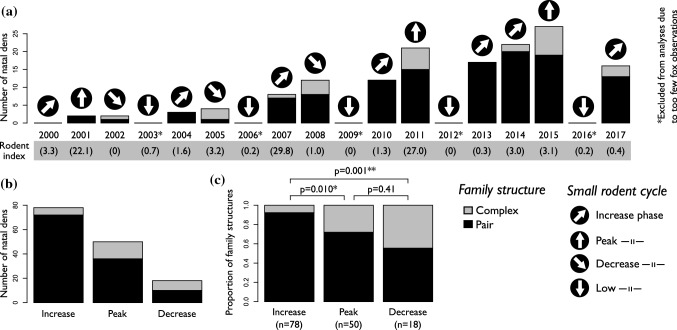
The number of arctic fox (*Vulpes lagopus*) dens with observed breeding (natal dens) 2000–2017. Arrows indicate phase of the small rodent cycle. Black represents reproducing pairs; grey represents complex family groups of 3 or more adults. 2b: Number of natal dens summed per small rodent phase. 2c: Proportion of complex families per small rodent phase. A pairwise Chi-square test showed that the proportion of complex families were lower during the increase phase compared to peak (*p* = 0.010) and the decrease (*p* = 0.001) phase, but that the latter did not differ from each other (*p* = 0.41)

The study was based on data collected between 2001 and 2017 in the area of Helagsfjällen in mid-Sweden (63° 00′ N, 12° 30′ E). The area consists of 3000 km^2^ mountain tundra with the birch forest tree line at about 900 m above sea level. All known dens (*n* = 63) in the core area were visited in July–August every year to detect reproduction (in total 149 litters recorded during 13 breeding years, Fig. 2). When a litter was identified, the dens were monitored to assess litter size, number of adults, identify ear-tagged adults, and to trap and ear tag cubs. When more than two adults were observed at the same den, it was classified as group-living. The number of visits and time spent at a den site typically varied between 2 and 3 times per season and lasting for on 2–5 days each, depending on over all workload, weather, and trapping success. Arctic foxes were trapped using baited Tomahawk live traps. The traps were under constant observation during trapping, and trapped individuals were ear-tagged (*Dalton rototag*), sexed, measured, and released immediately after trapping. During ear-tagging, a fragment of skin from the ear was collected for DNA analysis (see below). A maximum of 26 dens (41%) were occupied during a single year.

As a conservation measure, active dens in the study area were supplementarily fed *ad lib* with commercial dog food contained in feeding stations with small openings to prevent larger animals from entering (Angerbjörn et al. 2013). Feeding stations were installed within 150 m from den sites, primarily after a litter was born in the den for the first time (occasionally when den site was observed to be frequently visited) and kept active even if the den was deserted in following years. Supplementary food was always consumed but to a lower extent with increasing rodent abundance, indicating that foxes prefer natural prey (Thierry et al. 2020; personal observation). Since group-living has been shown to be more common in areas with frequent supplementary feeding (Elmhagen et al. 2013), we focused on the 146 litters with feeding and excluded three unfed litters from the analysis.

### Genetic detection of multi-parent litters

We used genetic parentage testing to identify potential cases of multiple or extra-pair paternity as well as cooperative breeding. In addition to visual observations of more than two adults at a den site, litters were classified as complex if genetic analyses showed that more than two parents were involved. For this, we assembled data on 11 polymorphic and autosomal microsatellite loci from a total of 733 individuals (148 litters) that were ear-tagged 2001–2017. The dataset included previously published data on 678 individuals (2001–2015, Norén et al. 2016; Hasselgren et al. 2018) and recently analysed data on 55 individuals (2016–2017). DNA storage, extraction, and PCR amplification were conducted following Hasselgren et al. (2018). Microsatellite alleles were size determined using LIZ-500 size standard (Thermo Fisher Scientific, Waltham, MA, USA) on an ABI3730 capillary sequencer (Applied Biosystems, Foster City, USA) at Macrogen Inc.

Parental assignment was performed using three approaches. First, the genotypes of any visually observed adults were manually compared with offspring genotypes (i.e., the exclusion method, Norén et al. 2016). A litter was classified as complex if there were more than four alleles present at a locus (multiple paternity, cooperative breeding), or if a litter had such genotype combinations that were not possible to inherit solely from two parents, or the visually observed adults (*n* = 1). Juveniles that were born at the same den during the same year but with divergent genotypes were then classified to belong to different litters (*n* = 15). Second, all genotypes were analysed in the software COLONY v. 2.0 (Jones and Wang 2010) following Hasselgren et al. (2018). Third, for litters with only one assigned parent, the genotype of the second parent was manually reconstructed and analysed in COLONY. We excluded 22 litters from the analyses, since there were too few juveniles ear-tagged to accurately determine the number of adults involved. Thus, the final dataset included 703 individuals from 126 litters. The probability of identity was approximately 1.0 × 10^–8^ for unrelated individuals and 3.0 × 10^–4^ for full-siblings (Hasselgren et al. 2018).

### Territories and geographical data

A typical arctic fox territory in Scandinavia would be about 25 km^2^ (i.e. about 2.8 km radius; Angerbjörn et al. 1997), containing a single den. In this study, however, we used a core area of 1 km radius centred around each den site. We believe that this approach allows a conservative comparison of the central foraging grounds. This 3.14 km^2^ core area was used for calculation of geographic parameters. Altitudinal data were retrieved from the Swedish mapping, cadastral, and land registration authority (*Lantmäteriet*) in 2 m resolution. Primary productivity was assessed by calculating normalized difference vegetation index (NDVI, Tucker 1979) from 10 m resolution LANDSAT-8 satellite images (acquisitioned 2015–08–19, pre-corrected for atmospheric distortion). We regard primary productivity as a proxy for prey abundance (per territory).

For calculation of NDVI, we used Quantum Geographic Information System (QGIS Development Team 2009). For calculation of per territory mean values, we used the Raster package (Hijmans and van Etten 2012) in R (R Core Team 2014).

A yearly index of small rodent abundance for the whole area was calculated based on snap trapping at six locations distributed across the study area. For each location, following the procedures of Krebs et al. (2002), we deployed 2 *Krebs lines* for 48 h that were checked every 12 h. Each Krebs line contained 60 bated snap traps in groups of three distributed evenly along a 300 m transect (Krebs et al. 2002; Le Vaillant et al. 2018). The total number of catches was summed up and presented as number of catches per 100 trap nights (in total 1440 trap nights per year). Based on the number of catches, and the change from the previous year, we classified the rodent abundance into four phases: increase, peak, decrease, and low phase (Fig. 2).

Birds of prey and carnivores that are strongly dependent on the small rodent cycle in northern regions (Nyström et al. 2006; Fuglei and Ims 2008; Hellström et al. 2014) switch to alternative prey during a rodent population crash. The arctic fox is one such alternative prey for the red fox (Frafjord et al. 1989; Tannerfeldt et al. 2002), golden eagle (Choi et al. 2019), and wolverine (Angerbjörn et al. 2004) where especially arctic fox cubs are targeted. Thus, the intra-guild predation pressure on the arctic fox is considered to follow the small rodent (basal prey) abundance (Nyström et al. 2006; Hellström et al. 2014; Larm et al. 2019), and we therefore used the small rodent phase as proxy for intra-guild predation pressure. We regarded intra-guild predation pressure to be highest during the decrease phase, and lowest during increase and peak phases (Table 4). We expected the lowest proportion of group-living during years when small rodents were undetectable. However, arctic foxes are notoriously difficult to observe when they do not breed and therefore spend little time at the den site. We were therefore unable to evaluate group-living during low rodent phases.

All animal trapping and handling were ethically evaluated and legally approved following Swedish legislation (permits number: 412-35-99 Nf, 412-4191-03 Nf, 412-5362-04 Nf, 412-7884-07 Nf, NV-01959-14, NV-02547-17, 30-1698/04, A65/99, A39-00, A49-01, A111-03, A74-05, A130-07, A131-07, A36-11, A36-11, A37-11, A18-14, A19-14).

### Statistical analysis

Based on the whole dataset spanning 2001–2017 (but excluding data from the 5 low years due to very limited field observations), we fitted the Full model: a linear mixed-effects model using a logit function and group-living as a binomial response variable. The following explanatory variables were included: small rodent phase (increase, peak and decrease), primary productivity (arithmetic mean NDVI per territory), mean altitude (km), distance to closest arctic fox neighbour (km), and small rodent index (catches per 100 trap nights). Altitude was used as a proxy for distance to the tree line which forms the boundary of the arctic fox habitat and increasing competition due to red fox presence (Hersteinsson and Macdonald 1992; Elmhagen et al. 2002; Herfindal et al. 2010). Distance to closest neighbouring den was included as individuals born in remote dens would have less chance to find an unrelated mate, and hence an increased incentive to become a joiner.

Arctic fox den ID was included as a random effect to control for the fact that several data points were recorded from the same territories during different years and sometimes concerning the same or closely related individuals. To evaluate if the Full model had any explanatory value, we compared it to a constant reference model (only containing the random factors) using Akaike Information Criterion (AIC, models that differed less than two were considered as equally supported).

Since we found a strong effect of small rodent phase on group-living, we made a pairwise comparison (Tukey method for *p* value adjustment) of the different small rodent phases to see how they differed from each other. While still presented in Fig. 2, we excluded data from the increase phase in all the following analyses as group-living families were few (< 8%). To investigate if there were different factors explaining group-living within phases, we fitted two specific models covering (a) the peak and (b) the decrease years of the small rodent cycle, respectively. Both models were identical to the Full model, except that small rodent phase was excluded as explanatory variable.

All statistical analyses were carried out in R (R Core Team 2014; RStudio Team 2015) using packages *CAR*, *Raster*, *LME4*, and *AICcmodavg* (Fox and Weisberg 2011; Hijmans and van Etten 2012; Bates et al. 2015; Mazerolle 2016).

## Results

Only a few litters with few cubs were born during low phases and data from those years were excluded (Fig. 2). Among the remaining 146 litters studied, we found group-living during the reproductive period in 28 (19%) cases. Most social groups consisted of three adults, but four adults were observed in three cases, and we made a single observation of six adults at the same den. We found a clear effect of fluctuations in prey abundance on group-living (*n*_obs_ = 146, *n*_groups_ = 33, degrees of freedom = 2, *p* < 0.002, Table 1) as the occurrence of group-living varied with small rodent phase (increase_pair_ = 72, increase_group_ = 6; peak_pair_ = 36, peak_group_ = 14; decrease_pair_ = 10, decrease_group_ = 8; Fig. 2). Pairwise comparison showed that the proportion of group-living during increase years was lower compared to both peak (*p* = 0.010) and decrease phase (*p* = 0.001). There was, however, no difference between peak and decrease phase (*p* = 0.41, Fig. 2c).

**Table 1:** The Full model explaining group-living in a Scandinavian Arctic fox population

	*p* values										
Model	Rodent phase	Primary productivity	Mean altitude	Dist. closest neighbour	Rodent index	*K*	AICc	ΔAICc	AICcWt	LL	Cum. Wt
*Full*	**0.002**	**0.030**	**0.049**	0.24	0.95	8	137.15	0.00	0.98	− 60.05	0.98
*Constant*						3	145.02	7.87	0.02	− 69.42	1.00

Based on all years of reproduction 2001–2017. The model performed better than the constant reference model (ΔAICc > 2)

Our prediction based on resource dispersion that group-living would be more common in more productive core areas, gained support as group-living increased with primary productivity (χ^2^ = 4.74, df = 1, *p* = 0.030) (Table 1). There was also a positive relationship between increasing altitude (used as a proxy for red fox abundance) and group-living (χ^2^ = 3.88, df = 1, *p* = 0.049). Distance to the closest neighbour, however, showed no effect (χ^2^ = 1.38, df = 1, *p* = 0.24), and the small rodent index had no additional effect beside the rodent phase (χ^2^ = 0.005, df = 1, *p* = 0.95).

The support for a positive effect of core area productivity was stronger when considering solely the data from the peak phase as group-living increased with primary productivity (n_obs_ = 50, n_groups_ = 29, χ^2^ = 6.19, df = 1, *p* = 0.013; Table 2a). A relationship between increased group-living and increasing distance to closest neighbour was suggested (χ^2^ = 3.63, df = 1, *p* = 0.057), as well as a positive effect of mean altitude (Full model: χ^2^ = 3.59, df = 1, *p* = 0.058). As in the analysis of the full data set, small rodent index did not show any effect (χ^2^ = 0.32, df = 1, *p* = 0.57).

**Table 2:** Specific models, explaining group-living in a Scandinavian Arctic fox population

	*p* values									
	Primary productivity	Dist. closest neighbour	Mean altitude	Rodent index	K	AICc	ΔAICc	AICcWt	LL	Cum. Wt
(A) Peak Phase										
Specific model	**0.013**	0.057	0.058	0.57	6	63.68	0.00	0.74	− 24.86	0.74
Constant					3	65.82	2.14	0.26	− 29.65	1.00
(B) Decrease phase										
Constant					3	32.44	0.00	0.99	− 12.37	0.99
Specific model	0.44	0.35	0.90	0.83	6	41.30	8.85	0.01	− 10.83	1.00

Based on data solely from (a) peak year and (b) decrease year of the small rodent cycle. The model of the peak phase performed better than the constant reference model (ΔAICc > 2). The decrease phase model did not perform better than the constant reference model, and we found no relationships explaining the occurrence of complex families

In the specific model for the decrease year, there was no relationship between group-living and any of the explanatory variables. The lack of any meaningful relationships was confirmed by that the constant reference model had a lower AIC (Table 2b).

### Relationship between cooperative breeding individuals

In addition to the visually observed 20 dens with group-living, 15 of 126 litters analysed through DNA were socially complex (detecting eight additional litters with more than one breeding female, Table 3). In 10 of these 15 cases, all parents involved could be successfully identified with tagged individuals. In nine of the litters, the group consisted of a mother and 1–2 of her daughters from previous years, which all reared litters together with 1–2 males in total. In most cases, the philopatric daughters were yearlings who subsequently dispersed as 2 years old to find their own territory. At one den site, however, the same mother and daughter remained for 3 consecutive years and reared litters together. We also identified one case of group-living consisting of two sisters from the same litter, and one male, where one of the sisters was the sole mother to all offspring (Table 3).

**Table 3:** Relationships in cooperative breeding Arctic fox families (*Vulpes lagopus*) that could be established through genetic analysis from tagged individuals

Year	Den ID	Group composition		Breeding females	Small rodent phase
		Females	Males		
2002	Den 20	♀♀	♂♂	Mother and 1 yearling daughter	Decrease
2005	Den 20	♀♀♀	♂♂	Mother and 2 yearling daughters	Decrease
2007	Den 33	♀♀	♂	Mother and daughter (born 2005)	Increase
2011	Den 19	♀♀♀	♂♂	Mother and 2 yearling daughters	Peak
2014	Den 19	♀♀*	♂	Mother and daughter (born 2010)*	Increase
2015	Den 19	♀♀*	♂	Mother and daughter (born 2010)*	Peak
2015	Den 20	♀♀	♂	Mother and 1 yearling daughter	Peak
2017	Den 29	♀♀	♂	Mother and daughter (born 2015)	Increase
2017	Den 09	♀♀**	♂	Mother**	Increase
2017	Den 33	♀♀	♂	Mother and 1 yearling daughter	Increase

*One of the daughters born in 2010 stayed in the territory

**Not cooperative breeding, the only female visually observed at the den was the sister of the mother of the litter

The three litters with multiple detected fathers showed large variation in relatedness. The two males in the first case descended from different pedigree founders (Norén et al. 2016) and were thus considered unrelated. In the second case, the two males were closely related (uncle–nephew), while one of the males in the last case was an immigrant from the Norwegian captive breeding program and hence completely unrelated to the other male.

## Discussion

We found that group-living in the arctic fox increased with territory quality and abundance of cyclic small rodents in accordance with the Resource Dispersion Hypothesis (Macdonald 1983). However, the group size remained high even when rodent populations crashed, i.e., when the main food resource largely disappeared, a pattern of inter-annual variation described as “the temporal emphasis of the Resource Dispersion Hypothesis” (Macdonald and Johnson 2015).

### Temporal resource variability

The increase in frequency of group-living between the increase rodent phase and the peak phase (from 8 to 28%, Fig. 2c, Table 4) supports the prediction that prey availability promotes the tolerance of accepting joiners. The prediction that the frequency of group-living would increase with core area productivity received support in the Full model (Table 1) and in the peak phase of specific models (Table 2), although the effect was weaker than the strong effect of small rodent phase (Table 1). Territories that support a resident pair and their litter during the small rodent increase or decrease phase could be expected to support additional adults during peak years. A similar pattern has been observed in Ethiopian wolves (*Canis simensis*) where increased juvenile survival together with high tolerance for joiners explained increased group size in high-quality territories (Tallents et al. 2012). Furthermore, the benefits accruing to yearlings that become joiners would increase with increasing territory quality if dispersal to a lower quality habitat lowers lifetime reproductive success (Macdonald and Carr 1989; Koenig et al. 1992).

**Table 4:** Predicted and observed effects of prey dynamics on family structure in 146 arctic fox (*Vulpes lagopus*) litters

Prey dynamics	Food abundance	Predation pressure	Predictions of the resource dispersion hypothesis	Cost–benefit of accepting joiners according to Hersteinsson’s model	Joiner perspective	Proportion complex families	Observation
Low year	Low	Low	Low tolerance to joiners	Low	[Unclear]	–	[Lack of data as non-breeding foxes are difficult to observe]
Increase year	Medium/high	Low	Moderate or high tolerance to joiners	Moderately positive (Moderate cost and low predation)	Low incentive to join	8% (*n* = 78)	Few litters with joiners, but candidate joiners are limited (few yearlings)
Peak year	High	Low	High tolerance to joiners	Moderately positive (Low cost, low predation)	Moderate incentive to join	28% (*n* = 50)	Prediction supported
Decrease year	High/low	High	Low tolerance to joiners, depending on timing	Positive (High cost, high predation)	Potentially strong incentive to join	44% (*n* = 18)	High proportion of litters with joiners, but few litters in total. No resolution to determine timing

Most herbivores in the tundra landscape, such as voles, passerines, and waders, are more common in more productive patches (Svensson et al. 1984; Oksanen et al. 1992, 1999; Elmhagen et al. 2002), while lemmings are more common in unproductive habitats during the increase phase (Le Vaillant et al. 2018). In highly productive territories, most relevant prey species would thus be expected to be more abundant during all phases but with less lemmings during the increase phase. This could be particularly important during the decrease phase when alternative prey becomes an important alternative.

There can also be marked changes in abundance within a year, especially when the rodent peak phase goes into a crash. This can happen during the fox breeding season (as in 2005, 2008, and 2015) or later (as in 2011). Our small rodent trapping method is unfortunately unable to detect such abrupt changes, which are by nature hard to quantify and detect also with extensive sampling, since changes does not have to appear uniformly and synchronous across the landscape (Oksanen et al. 1999).

### Intra-guild predation

Surprisingly, the proportion of fox families living in groups did not decline from peak to decrease phase (Fig. 2c). Since low prey availability would increase the cost for group-living, this result is not in straightforward accordance with the RDH. Instead, intra-guild predation pressure is strongly related to small rodent prey availability in the Scandinavian mountain tundra, and abrupt decreases of small rodent populations are typically associated with a prey switch among predators, resulting in increased intra-guild predation pressure on arctic fox from eagles, red foxes, and wolverines (Frafjord et al. 1989; Tannerfeldt et al. 2002; Larm et al. 2019). This negative relationship between resource abundance and intra-guild predation pressure reveals a trade-off between increased costs of sharing a territory and increased benefits of tolerating additional adults to take part in cooperative vigilance and defence. In an unpublished Master thesis (Isaksson 2012), we found a positive relationship between group size and the amount of time during which the cubs were guarded, and adult attendance has been linked to juvenile survival in the Scandinavian arctic fox (Erlandsson et al. 2017). Although an increase in rodent abundance is gradual, the decrease is often drastic (Turchin et al. 2000). The timing of such a crash varies, but it often happens during snow melt in the spring (Boonstra et al. 1998). Most carnivores and birds of prey in the mountain tundra ecosystem are highly dependent of small rodents with a peak in their reproduction during peak rodent years (Angerbjörn et al. 1995; Ims and Fuglei 2005; Hellström et al. 2014). When carnivores and birds of prey lose their rodent food resources in the middle of their reproductive season, they try to switch to alternative food (Nyström et al. 2006; Hellström et al. 2014). For the larger species, this switch includes arctic foxes as prey (Frafjord et al. 1989; Tannerfeldt et al. 2002; Larm et al. 2019). In the Scandinavian mountain, tundra arctic foxes lost entire litters to predation by golden eagles during a crash of small rodent populations (Choi et al. 2019). Thus, the increased intra-guild predation pressure on arctic foxes during the rodent crash years would increase the benefit of group-living despite growing scarcity of food resources. This would support the prediction of the Hersteinsson’s model where predation pressure would favour cooperative vigilance or defence and group-living (Norén et al. 2012). An alternative explanation would be that territories of residents are spatially configured to support joiners even during low food availability. However, in such cases, group-living would be far more common over all. Juvenile survival is generally high during increase and peak years (Meijer et al. 2013a), resulting in a surplus of candidate joiners.

It is rare to directly observe predation events and to measure rate of intra-guild predation. During 2015, the peak rodent year turned into a crash during early summer in another arctic fox subpopulation in Vindelfjällen, Sweden. Choi et al. (2019) observed 32 interactions between arctic foxes and their predators during the summer, mostly golden eagles (*n* = 25, 1 successful attack on an artic fox cub) and wolverines (*n* = 4) visiting fox dens. The lack of quantitative estimates of predation per se is a limitation of our study, but we argue that small rodent phase is a powerful proxy for intra-guild predation in this ecosystem. During 30 + years of fieldwork, we have observed that Scandinavian arctic foxes rely heavily on vigilant adults to warn juveniles of approaching threats, and in families with a single female or a pair, juveniles are more often left unattended (Isaksson 2012). We therefore regard the high proportion of group-living, despite declines in small rodent prey abundance, as support for the Hersteinsson’s model, and its emphasis on predation as a driver of group size (Norén et al. 2012). Consequently, the combination of variation in both resource availability and predation pressure would thus affect the tolerance that territorial residents show to joiners (as well as the reluctance of secondary group members to disperse), and thereby the tendency to form social groups.

### Other temporal and spatial effects

The effect of temporal resource dispersion, i.e., small rodent phase, is similar to the pattern observed in boreal red fox by Lindström (1982) and can be viewed from both a social and a demographic perspective, not necessarily mutually exclusive. Yearling arctic foxes comprised a higher percentage of cooperative breeders during peak and decrease years (Table 3). However, there were very few yearlings present during the increase phase, since a few cubs were born during the preceding low phase (Fig. 2a). The limited occurrence of group-living during increase years could hence reflect a lack of potential joiners due to a biased age structure and greater availability of vacant territories rather than an unwillingness of residents to tolerate group-living. The low den occupancy rate observed during our study suggests that there would be low competition for territories during increase years, as Scandinavian adult arctic foxes have a fairly high yearly mortality (0.28–0.40; Meijer et al. 2013b) and a few litters are born during low rodent years. In addition, Meijer et al. (2013b) found that, compared to cubs born during the decrease phase, the reproductive value of cubs born during the increase phase was 3.2 times higher. This was an effect of a generally high chance of surviving to adulthood and reproducing during the following peak phase, combined with a comparably low mortality as adults during the subsequent rodent decrease phase, also compared to individuals born during the peak that are yearlings during the decrease (Meijer 2013b). This suggests that adults with the potential to breed during increase years should strive to do so.

The reproductive value of offspring that have reached reproductive age is higher compared to younger offspring, and if joiners have an increased chance to survive compared to dispersers, residents might benefit from accepting adult offspring as joiners (Koenig et al. 1992). From a joiner perspective, the lower survival rate among cubs born to first time breeders observed in the arctic fox during decrease years (Erlandsson et al. 2017) suggests a high cost of breeding. Survival could potentially be higher with cooperative breeding (Erlandsson et al. 2017 did not consider litters raised by groups), but even a joiner that does not breed could gain inclusive fitness by improving survival rates of younger siblings (Emlen 1982), possibly in combination with benefits of acquiring breeding skills that would improve its future fitness (Brown 1987).

### Relatedness effects

Close relatives were more likely to form groups. All nine cooperative breeding female joiners that were detected through genetic analyses were adult daughters to the resident pair, and all but one was born in the previous year. In one group, a sister to the resident female was present at the den, but was not the mother of any of the captured cubs (Table 3). This general pattern suggests that inclusive fitness may be an additional factor influencing group formation and composition in the arctic fox. During peak rodent phases, there was a trend, suggesting that social groups may be more common in remotely located dens. In Scandinavian red foxes, related females were more closely associated in space compared to males and individuals of opposite sex (Walton et al. 2021), which is a likely consequence of male-biased dispersal and hence inbreeding avoidance. A similar pattern has also been observed in birds as Brouwer et al. (2011) saw that female red-winged fairywren (*Malurus elegans*) moved further away if they were closely related to neighbouring males. However, the probability for an arctic fox in our study population of finding an unrelated mate decreases with distance to neighbours and we expect that yearlings that do not find an unrelated mate are more prone to become joiners.

The cooperative breeding males, on the other hand, showed larger variation in relatedness. In the three genetically detected cases, we saw both closely related fathers (uncle–nephew) and completely unrelated individuals (a local fox and a captive bred immigrant). The relationship in the third case is unclear, since they originated from two different founders (Godoy et al. 2018) and, as a consequence, are assumed to be unrelated. The completely unrelated fathers were observed coexisting at the den for 4 days without any signs of hostility towards each other (personal observation). If female joiners are related to the territorial pair, unrelated male joiners would then minimize the potential for inbreeding.

Despite the generalisation that primary productivity decreases with elevation, we saw indications that group-living increased with altitude (Tables 1, and 2a). This suggest that the high-quality arctic fox territories are characterised by relatively high primary productivity, while still remaining pure mountain tundra habitat. The distributions of cold adapted animals are usually limited by competition rather than by climate per se (MacArthur 1984; Hersteinsson and Macdonald 1992). The birch forest and the tree line form the southern limit of the arctic fox distribution range in Scandinavia and the forest is a more favourable habitat for boreal species such as the larger red fox. From a territory defence perspective, residents should be more willing to accept joiners if there is a perceived threat of predation, parallel to the regional differences described in Hersteinsson’s model (Norén et al. 2012; Table 4). We could therefore expect an increase in group-living at lower altitudes as a response to increased inter-specific competition and intra-guild predation. In previous studies, however, arctic foxes were less likely to breed in dens closer to the tree line (Elmhagen et al. 2002; Dalén et al. 2004), suggesting that when establishing territories, the response of the arctic fox to perceived stress is to move rather than increase group size (Elmhagen et al. 2013).

### Supplementary feeding

The observed differences in group-living related to small rodent phase and core primary productivity might be unexpected insofar as all litters in the study have been provided with supplementary food. Considering that group-living has been observed to increase in supplementary fed populations (Elmhagen et al. 2013), the number of group-living social units that we observed is probably higher than it would have been in an unfed population. We conclude that supplementary food is increasing the tendency of group-living, but not equating that tendency amongst all the territories. Foxes prefer natural food items and consume less supplementary food at high small rodent abundance. The strong connection between number of litters and litter size also in supplementary fed populations, strikingly exemplified by the very limited number of reproductions observed during small rodent low years, indicates that foxes remain sensitive to natural cues and that they utilise, but do not depend on, the supplementary food.

### Concluding remarks

Animals exposed to cyclic variation in resources often display a range of physical and behavioural adaptations. Studying their ability to respond to fluctuations in food abundance on multiple levels can be informative as well as challenging. We took advantage of a study system exposed to inter-annually and seasonally variable resource abundance and intra-guild predation pressure to study the mechanisms of group-living. Our results showed an effect of territory quality on the occurrence of group-living, supporting the RDH. This effect did, however, disappear during the small rodent decrease phase, when predation rather than resource availability appeared to be more important. We suggest that the ideas of both Hersteinsson (predation) and Macdonald (temporal resource dynamics) are needed to accurately explain group-living among prey species and mesopredators. The “Hersteinsson-Macdonald dynamics” would likely be especially apparent in ecosystems with cyclic basal prey fluctuations.

## Data Availability

The arctic fox is a protected species in Scandinavia, and to not disclose the location of arctic fox dens, the geographic data analysed in this study cannot be provided for public. However, genetic data are available on request.

## Code availability

Code without data is available on request.
